# Diagnostic accuracy of the Xpert^®^ MTB/XDR assay for detection of Isoniazid and second-line antituberculosis drugs resistance at central TB reference laboratory in Tanzania

**DOI:** 10.1186/s12879-024-09562-z

**Published:** 2024-07-04

**Authors:** Togolani Maya, Aman Wilfred, Clara Lubinza, Saidi Mfaume, Maryjeska Mafie, Daphne Mtunga, Amri Kingalu, Nicodem Mgina, Pammla Petrucka, Basra E. Doulla, Esther Ngadaya, Sayoki G. Mfinanga, Nicholaus P. Mnyambwa

**Affiliations:** 1 Central TB Reference Laboratory, Dar es Salaam, Tanzania; 2 Muhimbili Research Centre, National Institute for Medical Research, Dar es Salaam, Tanzania; 3 College of Nursing, University of Saskatchewan, Saskatoon, Canada; 4 Alliance for Africa Health and Research, Dar es Salaam, Tanzania; 5 Kampala International University, Dar es Salaam, Tanzania

**Keywords:** Xpert MTB/XDR, Tuberculosis (TB), Drug-resistant TB, Line Probe Assay (LPA), Diagnosis

## Abstract

**Introduction:**

Early diagnosis of tuberculosis (TB) and universal access to drug-susceptibility testing (DST) are critical elements of the WHO End TB Strategy. Current rapid tests (e.g., Xpert^®^ MTB/RIF and Ultra-assays) can detect rifampicin resistance-conferring mutations, but cannot detect resistance to Isoniazid and second-line anti-TB agents. Although Line Probe Assay is capable of detecting resistance to second-line anti-TB agents, it requires sophisticated laboratory infrastructure and advanced skills which are often not readily available in settings replete with TB. A rapid test capable of detecting Isoniazid and second-line anti-TB drug resistance is highly needed.

**Methods:**

We conducted a diagnostic accuracy study to evaluate a new automated Xpert MTB/XDR 10-colour assay for rapid detection of Isoniazid and second-line drugs, including ethionamide, fluoroquinolones, and injectable drugs (Amikacin, Kanamycin, and Capreomycin). Positive Xpert MTB/RIF respiratory specimens were prospectively collected through routine diagnosis and surveillance of drug resistance at the Central TB Reference Laboratory in Tanzania. Specimens were tested by both Xpert XDR assay and LPA against culture-based phenotypic DST as the reference standard.

**Findings:**

We analysed specimens from 151 TB patients with a mean age (SD) of 36.2 (12.7) years. The majority (*n* = 109, 72.2%) were males. The sensitivity for Xpert MTB/XDR was 93.5% (95% CI, 87.4–96.7); for Isoniazid, 96.6 (95% CI, 92.1–98.6); for Fluoroquinolone, 98.7% (95% Cl 94.8–99.7); for Amikacin, 96.6%; and (95% CI 92.1–98.6) for Ethionamide. Ethionamide had the lowest specificity of 50% and the highest was 100% for Fluoroquinolone. The diagnostic performance was generally comparable to that of LPA with slight variations between the two assays. The non-determinate rate (i.e., invalid *M. tuberculosis* complex detection) of Xpert MTB/XDR was 2·96%.

**Conclusion:**

The Xpert MTB/XDR demonstrated high sensitivity and specificity for detecting resistance to Isoniazid, Fluoroquinolones, and injectable agents. This assay can be used in clinical settings to facilitate rapid diagnosis of mono-isoniazid and extensively drug-resistant TB.

## Introduction

Tuberculosis (TB) is one of the leading causes of mortality and morbidity particularly in low and middle-income countries [1]. Millions of people continue to fall sick with TB each year. The situation is exacerbated by the emergence and spread of drug-resistant TB. At least 450 000 develop rifampicin-resistant/multidrug-resistant TB (RR/MDR-TB) globally each year [1], of which about 10% are classified as extensively drug-resistant (XDR-TB) [1, 2]. MDR-TB includes resistance to both Isoniazid and Rifampicin, whereas pre-XDR is an MDR-TB that extends resistance to at least one fluoroquinolone [3]. Recently, the World Health Organization (WHO) redefined XDR-TB as an MDR/RR-TB that is resistant to a fluoroquinolone and/or Bedaquiline or Linezolid [3]. RR-TB may be in conjunction with or without resistance to any other anti-TB drug [4].

Effective treatment is possible if TB-infected persons are detected early and put on TB treatment immediately. The WHO End TB Strategy calls for universal access to TB drug susceptibility testing (DST) to ensure that the most effective treatment regimen is prescribed as early as possible [5]. Global coverage of testing for resistance to second-line anti-TB drugs remains much lower, with only 50% of all bacteriologically confirmed TB cases being tested for resistance to fluoroquinolones [1]. To realize the goals of the End TB Strategy, point-of-care diagnostic tools that not only offer high sensitivity and specificity but also generate results rapidly are highly required. Culture and phenotypic drug susceptibility testing (pDST) of MTB are time consuming, labor-intensive, and present a serious biohazard to laboratory workers, resulting in fewer facilities in countries where TB is endemic. Even when available, culture-based DST has a long turn-around time which renders it impractical to use for routine diagnostic purposes. The development of the GeneXpert MTB/RIF assay (Cepheid, Sunnyvale, USA) was a major step forward in improving the diagnosis of TB and Rifampicin resistance detection globally [6]. However, Xpert MTB/RIF assay and its improved generation, ultra-assay, detect only rifampicin resistant conferring mutations, while continuing non-detection of resistance to Isoniazid and second-line anti-TBs, such as Fluoroquinolones. Isoniazid and Rifampicin are two most powerful first-line anti-TB drugs, and resistance to either of these increases the risk of treatment failure, relapse, or acquisition of resistance to other drugs. The only available WHO-recommended rapid molecular test for the detection of strains resistant to second-line anti-TB agents, HAIN Line Probe Assay LPA (MTBDR*sl*) [7] requires sophisticated laboratory infrastructure, equipment and skilled personnel [8, 9]. Hence the need for a rapid drug-susceptibility test that can detect resistance to the most common first and second-line drugs, and that requires low technical skills and infrastructure.

Cepheid recently developed a new TB cartridge known as Xpert MTB/XDR assay with in-built system of quality controls that can detect resistance-conferring mutations to Isoniazid (*inhA* promoter, *katG*, *fabG1*, oxyR-aphC intergenic region), and second-line drugs: ethionamide (*inhA* promoter), Fluoroquinolone resistance-associated mutations in the *gyrA* and *gyrB* quinolone resistance determining regions (QRDR), and second-line injectable drugs (Amikacin, Kanamycin, and Capreomycin) associated mutations in the *rrs* gene and the *eis* promoter region [10, 11]. The assay is an automated in vitro diagnostic test for detection of *M. tuberculosis* DNA and resistance associated mutations. The test is performed on Cepheid GeneXpert Instrument Systems equipped with 10 color modules. The Xpert instrument systems integrate and automate sample processing, nucleic acid amplification, and detection of the target sequences in samples using nested real-time PCR and melt peak detection [10, 11]. The assay has the same sample processing and workflow as that of the Xpert MTB/RIF. The primary objective of this study was to estimate the diagnostic accuracy of the novel GeneXpert XDR assay against pDST as the gold standard and further compare its performance with that of LPA.

## Methodology

### Study design and settings

This prospective diagnostic accuracy study was conducted at the Central TB Reference Laboratory (CTRL) in Tanzania. Samples for this study included those received for routine diagnosis of TB and DR-TB from various health facilities within Dar es Salaam. We also included positive sputum samples collected through routine surveillance of drug resistant TB, from facilities located in Dar es Salaam. At the health facilities TB was bacteriologically diagnosed by Xpert MTB/RIF. At CTRL specimens were tested by Xpert MTB/XDR assay and LPA to evaluate resistance to Isoniazid, ethionamide, fluoroquinolones (Ofloxacin) and second-line injectables drugs (Amikacin, Kanamycin and Capreomycin) with pDST as the reference standard. Specimens positive for pulmonary TB (susceptible and rifampicin resistant TB cases) were consecutively analyzed as they reach at the CTRL, between January and April 2022. Laboratory technicians received training on the new technology prior to commencing testing.

### Laboratory procedures

Direct sputum specimens were processed for the Xpert MTB/XDR assay while concentrated sediments prepared from sputum were used to perform LPA and culture-based pDST assays. Xpert MTB/XDR and LPA were performed in accordance with the manufacturer’s instructions. Xpert MTB/XDR assays were performed on the Xpert Instrument Systems upgraded with 10 colour technology donated by Cepheid. Phenotypic DST for the first and second anti-TB drugs was performed in a contained BSL 3 facility as per the WHO recommendations using Lowenstein-Jensen (LJ) media slants or liquid mycobacteria growth indicator tubes (MGIT) [12]. In summary, for LJ bacterial isolates were inoculated onto the LJ and incubated at 35-37^o^C until growth was observed or discarded as negative after 8 weeks incubation. Cultures with positive results were tested for pDST against respective drugs. MGIT was used to evaluate resistance to Kanamycin, Ofloxacin, Levofloxacin, Ethionamide and/or Amikacin. All pDST assays were performed in triplicate to ensure reproducibility.

### Statistical analysis

Demographic characteristics of the participants (i.e., age, sex, residence, and occupation) and clinical information (HIV status and TB treatment history) were extracted from the TB lab Information system at CTRL. The data dictionary was developed in MS Excel in which the Xpert MTB/XDR results were added to get the complete dataset. Data analysis was carried out using STATA version 12. Categorical variables were described as proportions or frequencies while continuous variables presented as means and standard deviations, or range. Diagnostic performance characteristics were analyzed as sensitivity, specificity, Positive Predictive Value (PPV), Negative Predictive Value (NPV), with their respective 1-α confidence intervals. Culture pDST was used as the reference standard in this analysis.

## Results

### Demographic and clinical characteristics of study participants

**Fig. 1:**
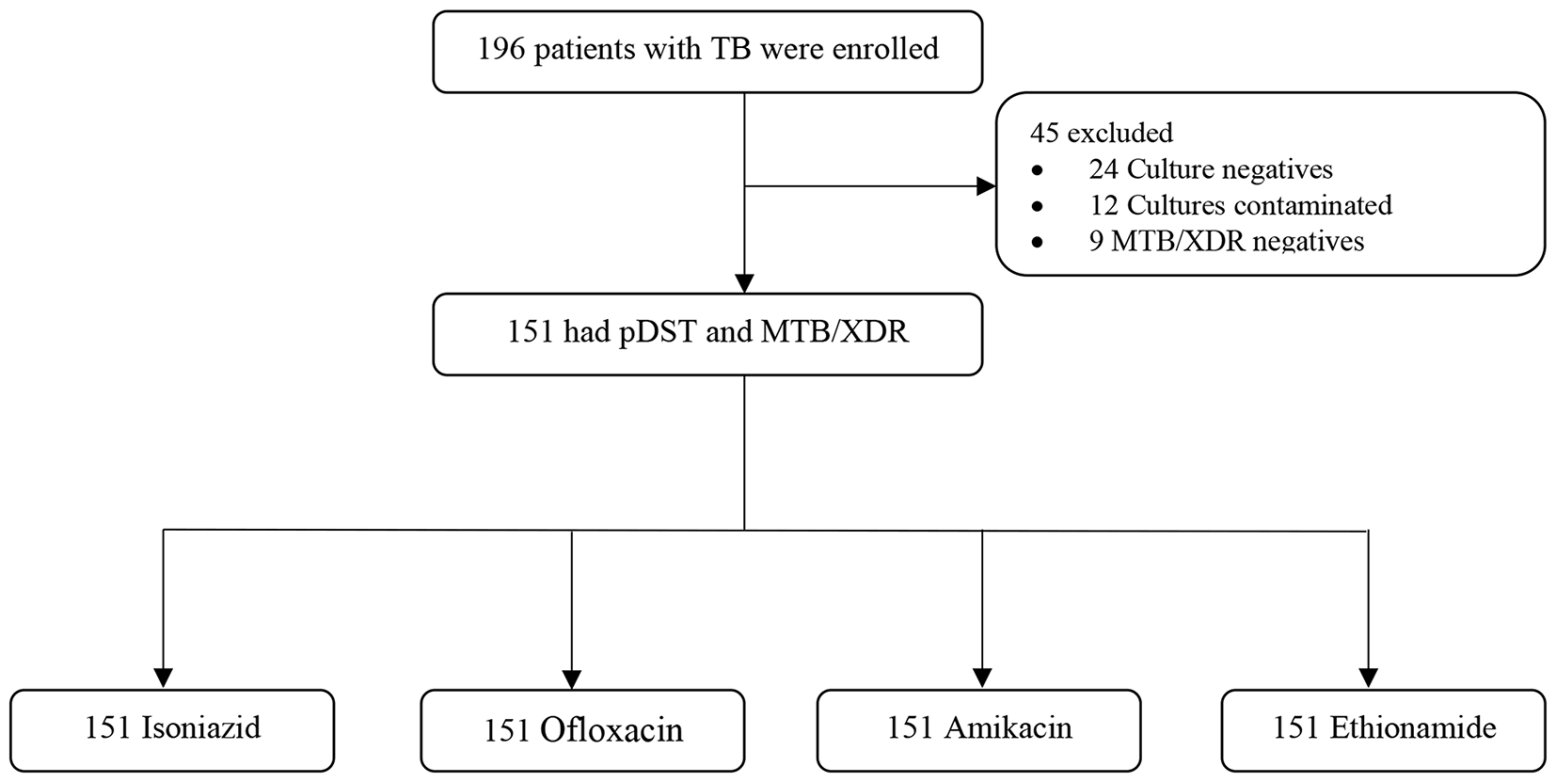
Participants enrollment and exclusion

Between January and April 2022, 196 patients with TB were enrolled, but 45 were excluded because they were either negative on culture, contaminated or TB not detected by MTB/XDR; hence, rendering evaluation of their drug susceptibility profiles impossible (see Fig. 1). All 151 specimens had paired DST results (pDST vs. MTB/XDR DST) for the four drugs: Isoniazid, Fluoroquinolone (Ofloxacin), Amikacin, and Ethionamide.

The analysis consisted of 151 patients with mean (SD) age of 36.2 (12.7) years. Among 151 patients, 109 (72.2%) were males. Based on the treatment history, 27 (17.9%) were patients with previously treated TB while 21 (13.9%) participants were HIV seropositive. Thirty-one (20.5%) participants had Rifampicin resistant strains detected by Xpert MTB/RIF at peripheral health facilities (Table 1).

**Table 1:** Demographic and clinical characteristics of study participants, *N* = 151

Variable	*N* (%)
**Age (years)** mean (SD)*	36.2 (12.7)
**Age categories**	
0–14	4 (2.5)
15–54	143 (89.4)
≥ 55	12 (7.5)
Missing	1 (0.6)
**Gender**	
Male	109 (72.2)
**HIV status**	
Seropositive	21 (13.9)
Seronegative	109 (72.2)
Unknown	21 (13.9)
**Patient category**	
New TB cases	118 (78.2)
previously treated TB cases	27 (17.9)
Follow-up	6 (4.0)
**Peripheral Xpert MTB/Rif**	
Rifampicin TB resistant	31 (20.5)
Rifampicin TB susceptible	120 (79.5)

*Age for one participant was missing

### Drug susceptibility profiles on MTB/XDR assay

The drug susceptibility profile for the 151 patients is provided in Table 2. Two patients had RR-TB and Fluoroquinolone resistance but susceptible to injectables (pre-XDR cases). The rate of indeterminate ranged from 0.6 to 3.1%. There were only two Isoniazid mono-resistant cases detected by both culture and XDR/MTB. The percentage of non-determinate (error/invalid) on MTB/XDR assay was 2.5% (*n* = 4) specimens. When repeated the assay, the results were valid for all 4 specimens.

**Table 2:** Test results for each drug on the Xpert MTB/XDR Test, *N* = 151

Variable	*n* (%)
**Isoniazid**	
Low resistance	2 (1.3)
Resistance detected	31 (20.5)
Susceptible	117 (77.5)
Indeterminate	1 (0.6)
**Fluoroquinolone**	
Low resistance	4 (2.7)
Resistance detected	2 (1.3)
Sensitive	143 (94.7)
Indeterminate	2 (1.3)
**Amikacin**	
Intermediate	2 (1.3)
Resistance not detected	149 (98.7)
**Kanamycin**	
Intermediate	3 (2.0)
Resistance not detected	148 (98.0)
**Ethionamide**	
Resistance detected	5 (3.3)
Resistance not detected	145 (96.0)
Indeterminate	1 (0.7)
**Capreomycin**	
Resistance detected	3 (2.0)
Resistance not detected	148 (98.0)

### Diagnostic performance of MTB/XDR

The diagnostic accuracy of MDR/XDR assay is provided in Table 3. The sensitivity was greater than 93% with the highest being 98.7% for Amikacin. Ethionamide had the lowest specificity of 50.0%.

**Table 3:** Diagnostic accuracy of the MTB/XDR against culture as gold standard

Drug	*N*	Sensitivity (%)	Sensitivity (95%CI)	Specificity (%)	Specificity (95%CI)	PPV (%)	PPV (95%CI)	NPV (%)	NPV (95%CI)
**Isoniazid**	151	93.5	87.4–96.7	92.9	73.7–98.3	98.3	93.3–99.6	76.5	58.5–88.2
**Fluoroquinolone**	151	96.6	92.1–98.6	100	-	100	-	65.5	58.7–79.2
**Amikacin**	151	98.7	94.8–99.7	*		100	-	*	-
**Ethionamide**	151	96.6	92.1–98.6	50.0	**	99.3	95.2–99.9	46.7	28.9–81.4
**Kanamycin**	151	98.0	93.9–99.4	*	-	100	-	*	-

‘*’ All observations were culture positive, hence specificity/NPV could not be determined. ** Too wide confidence interval due to small sample size (**-)** confidence interval could not be determined because specificity/PPV/NPV was 100%. We did not have pDST results for Capreomycin because it is no longer in use in Tanzania; hence, it was excluded

### Xpert MTB/XDR vs. LPA

The Xpert MTB/XDR assay diagnostic performance are comparable to that of LPA with slight variations. Xpert MTB/XDR had slightly higher sensitivity for Isoniazid while slightly lower for Fluoroquinolone, Kanamycin, and Amikacin resistance (Table 4).

**Table 4:** Comparison of performance for Xpert MTB/XDR in sputum and LPA using pDST as gold standard

	*N*	TP	FP	FN	TN	Sensitivity	Specificity	PPV	NPV
Isoniazid resistance									
MTB/XDR	148	112	2	8	26	93.3 (87.1–96.7)	92.9 (73.7–98.4)	98.3 (93.3–99.6)	76.5 (58.5–88.2)
MTBDR*plus*	148	111	1	9	27	92.5 (86.1–96.1)	96.4(76.3–99.6)	99.1 (93.8–99.9)	75.0 (57.6–86.9)
**Fluoroquinolone resistance**									
MTB/XDR	147	140	0	2	5	96.6 (92.1–98.6)	100 (-)	100 (-)	71.4 (64.2–78.5)
MTBDRsl	147	144	0	2	1	99.3 (95.2–99.9)	100 (-)	100 (-)	66.7 (53.1–99.9)
**Amikacin resistance**									
MTB/XDR	147	145	0	2	0	98.7 (94.8–99.7)	*	100 (-)	*
MTBDRsl	147	147	0	0	0	100 (-)	*	100 (-)	*
**Kanamycin resistance**									
MDR/XDR	147	144	0	2	0	98.0 (93.8–99.3)	*	100(-)	*
MTBDRsl	147	147	0	0	0	100 (-)	*	100(-)	*

‘*’ All observations in culture were sensitive, hence specificity could be determined. (-) means that the confidence interval could not be determined because specificity/PPV/NPV was 100%. Analysis was limited to matched patients with results from both an Xpert MTB/XDR and LPA assays to ensure direct comparison

## Discussion

In this study, we found high diagnostic accuracy of the MTB/XDR assay for detecting resistance to Isoniazid, Fluoroquinolone, Kanamycin, Amikacin, and Ethionamide among patients with pulmonary TB. The sensitivity for all five drugs evaluated against culture was above 93% with the highest being 98.7% for Amikacin. The assay also demonstrated high specificity for Isoniazid (92%) and Fluoroquinolone (100%). These findings are in line with a recent clinical trial conducted by the Foundation for Innovative New Diagnostics [10]. In this multicounty clinical study, the sensitivity varied significantly across drug type and study sites. For example, while the overall sensitivity for Isoniazid was 94%, the performance by site ranged from 80 to 99%.

We report high specificity for Isoniazid and Fluoroquinolone, which agree with the previous reports [10, 13], but low as 50% specificity for Ethionamide. The previous study reported the opposite, low sensitivity Ethionamide resistance but relative high specificity [10]. While multiple genes have been reported to cause resistance to Ethionamide [13], XDR/MTB assay detects resistance in only one gene region, the *inhA* promoter, and this may contribute to such variation. We could not determine the specificity for Amikacin and Kanamycin because all specimen were drug susceptible in the reference standard and this can be attributed to a small sample size used in this validation. Furthermore, differences in local populations, laboratories, and *M. tuberculosis* strains can explain such variations in the diagnostic performance of the assay [10]. A matched comparison of the results from Xpert MTB/XDR and LPA assays against the reference standard demonstrated high sensitivity and specificity that ranged from 92 to 100% for both assays. The XDR/MTB had slightly high sensitivity for Isoniazid (93.3%) as compared to 92.5% for LPA MTBDR*plus* [10]. Similar performance pattern was observed in the previously multi-country study and attributed to addition of two gene targets-*faBG* and intergenic region [10]. By contrast, the LPA MTBDR*sl* showed slightly higher sensitivity for Fluoroquinolone, Amikacin and Kanamycin than that of MTB/XDR [10], suggesting potential for further implementation assessment in different settings [8]. Our findings affirmed low rate of indeterminate (0.6–3.1%) and non-determinate (2.5%) results as previously reported [10].

The Xpert MTB/XDR test is easy-to -use and offers DST results in less than 2 h with minimal staff training and biosafety/infrastructure requirements compared to other WHO-endorsed molecular diagnostics for DR-TB agents [7]. The assay relies on melt curves that allow for the differentiation between wild type and mutant sequences and detects multiple mutations across several genes from a single specimen [10, 11], while the closed cartridge system minimizes risk of contamination. The assay uses existing Xpert system, allowing placement of the system at lower-level health facilities. This makes the assay ideal choice for detection of Isoniazid and Fluoroquinolones resistance for better patient outcomes [8]. However, the existing Xpert instrument will require upgrade of modules with 10 colour multiplex technology that may pose challenge in resources limited countries. In this evaluation, XDR/MTB correctly identified two cases of Isoniazid mono-resistance TB and two pre-XDR-TB cases. Isoniazid mono-resistant and pre-XDR TB requires customized regimens and hence underscore the added value of XDR/MTB assay for early detection and timely initiation of appropriate therapy. Although case definition of XDR-TB was recently updated [3], rapid detection of mutations associated with Fluoroquinolone and Isoniazid resistance is critical for preventing further resistance and improving clinical outcomes. We recommend the initial rollout of the assay at national reference or zonal laboratories. It should be used primarily as a reflex test for bacteriologically-confirmed TB, complementing existing rapid tests that detect only Rifampicin resistance. Additionally, more evidence on performance characteristics in different populations and a cost-benefit analysis would be valuable.

### Limitations

We could not assess the assay’s performance in paucibacillary disease in smear-negative patients and those living with HIV due to the small sample size, which may impact diagnostic efficacy. Samples were also obtained from a limited geographical area, so the results cannot be generalized to the entire country. We did not perform genome sequencing of the isolates, so discordant results between tests cannot be ruled out, however, the high diagnostic accuracy demonstrated by both assays is reassuring.

## Conclusion

This study provides in-country validation evidence on the diagnostic accuracy of the XDR/MTB assay for rapid evaluation of resistance to Isoniazid, Fluoroquinolones, and injectable agents for patients with bacteriologically confirmed pulmonary TB. Additional data on the cost-effectiveness and feasibility of implementing the Xpert MTB/XDR assay in various healthcare settings would be valuable.

## Data Availability

The datasets used and/or analyzed during the current study available from the corresponding author on reasonable request.
